# Focal Therapy in Grade Group 3 Prostate Cancer

**DOI:** 10.1007/s11934-024-01211-x

**Published:** 2024-07-02

**Authors:** Lee Pressler, Mariel Pressler

**Affiliations:** 1 Clinical Instructor Rutgers New Jersey Medical School, Morristown Medical Center, Morristown, NJ USA; 2 New York Columbia Presbyterian Medical Center, New York, NY USA

**Keywords:** Focal Therapy, Prostate cancer, Intermediate risk, HIFU, Cryotherapy, IRE

## Abstract

**Purpose of this Review:**

Treatment of intermediate risk prostate cancer remains controversial. Clearly some patients with low volume favorable intermediate risk can be followed with active surveillance. Those with high volume bilateral disease need more radical whole gland therapy. The question remains on how to best treat low volume localized unfavorable intermediate risk prostate cancer (GG3) while maintaining quality of life. Focal therapy has been becoming a popular option for many patients with localized prostate cancer. Most studies looking at focal therapy for prostate cancer have been limited to GG1 and GG2, many of whom may not need treatment. We set out to review the literature evaluating the safety and efficacy of focal therapy for GG3 prostate cancer.

**Recent Findings:**

We reviewed multiple peer review articles obtained from a PubMed search. While in field biopsy recurrence rates approach 20%, failure free survival and overall survival exceeds 90%.

**Summary:**

While focal therapy for unfavorable GG3 intermediate risk prostate cancer may have higher rates of local recurrence with appropriate post procedure follow up, patients who need salvage therapy are easily identified and survival rates are very high. Focal therapy is a good option for patients with localized low volume GG3 prostate cancer without compromising cancer survival and preserving quality of life.

## Introduction

Prostate cancer is the leading cause of cancer in men over 50 years old. With the wide use of screening since the late 1980’s there has been a large increase in the number of men diagnosed with early prostate cancer. The shifting of time of diagnosis to as many as 10 years earlier has resulted in a significant decrease in prostate cancer related deaths over the past 30 years. However, this has come at the cost of over treating many men with low risk cancer resulting in often life changing side effects such as erectile dysfunction, stress urinary incontinence and radiation related injury. 

With the increased use of active surveillance for low risk grade group 1 (GG1) and some grade group 2 cancers (GG2) the pendulum has swung back to a more appropriate place whereby we tend to observe low risk patients and treat high risk patients. The controversy remains on how to treat patients with intermediate risk prostate cancer, specifically those with unfavorable intermediate risk prostate cancer with grade group 3 Gleason 7 (4 + 3) disease. Do these patients need whole gland radical therapy with its associated quality of life issues or is there a subset of patients who could be safely treated with a less invasive modality coupled with close monitoring?

While radical prostatectomy or radiotherapy remain the standard of care for localized intermediate risk prostate cancer, concerns about overtreatment and side effects have driven the exploration of minimally invasive alternatives. Focal therapy (FT) targets only the cancerous foci within the prostate, aiming to preserve healthy tissue and potentially minimize morbidity. The wide use of mpMRI has enabled clinicians to better map the location of prostate cancer within the prostate and often identify an index lesion that may be the driver of the cancer’s clinical activity. Radtke et al. looked at radical prostatectomy specimens and found that 96% of the index lesions were correctly identified with the combination of MRI fusion and saturation biopsies [1]. 

As a result, we have been able to identify several groups of patients who are possible candidates for focal therapy. Specifically, patients with GG2-3 only in the suspicious MRI lesion. Patients with high grade cancer on one side, and lower grade cancer on the opposite side for which active surveillance (AS) would be appropriate. Lastly, patients with cancer only on one side of the prostate that would be a candidate for hemi-ablation.

There has been a lot of literature evaluating focal therapy for GG1 and GG2 prostate cancer with most patients in studies being GG1 patients. There have been very few studies focusing on GG3 patients. Unfortunately, most studies including GG3 patients have only a handful of cases and often all intermediate grade tumors are lumped together and GG3 outcomes are not reported separately. This review evaluates the existing peer-reviewed literature on the use of FT for GG3 prostate cancer, focusing on oncological outcomes, functional preservation, and safety.

## Results

### Oncologic Outcomes

#### High Intensity Focused Ultrasound (HIFU)

Stabile et al. evaluated 1032 men treated with focal HIFU. 15.6% and 1.4% were GG3 and GG4 respectively. Failure Free Survival (FFS) for GG3 was 77.8% vs. 86.5% for GG2 at 24 months and 37.4% for GG3 and 60.5% for GG2 at 60 months. Despite the increased need for retreatment (either repeat focal or salvage whole gland therapy) in GG3 patients overall survival for the entire cohort at 96 months was very high at 96.6% [2]. While the authors discouraged focal therapy for GG3 or higher we feel for a properly selected patient with low volume disease and who can have close follow up w repeat serial MRI and biopsy it is safe to treat these patients focally with the knowledge that they have increased risk of needing re-treatment in the future without compromising survival while sparing the majority of patient the need for whole gland radical therapy and its associated risks [2, 3]. 

Guillaumier et al. looked at 625 consecutive patients undergoing HIFU focal therapy. 86 patients (14%) were GG3. 5 year Kaplan Meier estimates for intermediate risk patients for overall survival, Metastasis free survival, failure free survival were 99%, 99%, 88% respectively. FFS for Gleason 7 (all 7) and Gleason 8 at 5 years was 87% and 59% [4]. In this patient population HIFU appears to be a safe and effective treatment option for properly selected group of GG3 patients.

#### Cryotherapy

A 2023 single center study of 163 patients of which 26 were GG2/3 and 15(70.5%) were GG 4/5(14.1%) reported 5-year BCRFS rates of 74% and 55% for GG3/4 and GG4/5 respectively. One problem with the study is that there was no post op biopsy performed. Biochemical recurrence may not be a good surrogate for cancer control. They also did not look at cancer specific or overall survival. Many studies show mixed results using PSA. It’s clear that repeat MRI and surveillance biopsy is the gold standard for monitoring patients after focal therapy [5]. 

A 2019 review evaluated medium term outcomes for focal cryotherapy for intermediate risk and high risk cancers. They looked at 122 consecutive patients from 2013 to 2016. Median follow up was 27.8 months. 35 patients had high risk cancer and 87 patients had intermediate risk cancer. 19 patients had GG3 and 2 patients had GG4 cancer respectively. Overall failure free survival at 3 years was 90.5%. 84.7% for high risk and 93.3% for intermediate risk cancer [6]. 

#### Irreversible Electroporation

Irreversible electroporation (IRE) is a novel focal therapy modality which utilizes pulsatile electrical currents to ablate tissue. The mechanism in which IRE does this is through the destabilization of the cell membrane, causing the alteration of membrane shape and the formation of nanopores. The excessive permeability of these cells disrupts the osmotic balance, leading to irreversible damage and the process of apoptosis [7]. 

Yaxley reported a cohort of 64 men treated with IRE, 15 of which had GG3 cancer. Median follow up was 23 months. 12 patients had GG4 or GG5 cancer. The median PSA nadir in this cohort was 1.3 ug/L (range 0.07–7.20 ug/L). The PSA nadir ranged from 1.10 to 114.55% of the original pre-IRE PSA level, with a median of 26.5% [8]. 

Complete ablation of all in-field cancer was identified in 35/40 (87.5%) of the surveillance biopsies. 80% of the cohort had follow up biopsies at about 1 year. Significant in-field recurrence was identified in 3/40 (7.5%) of surveillance biopsies, or 4/40 (10.0%) using definition 2 of any ISUP 2 or greater. Three patients with significant in-field recurrence have since proceeded to robot assisted laparoscopic radical prostatectomy (RALP). Of the two that had been done at time of publication, they had Gleason 3 + 4 and 4 + 3 respectively. Both patients were staged as pT2 with negative margins from their RALP histopathology. The fourth elected on a conservative approach, due to a minimal component of GG2 malignancy. Of the four in-field recurrences with GG ≥ 2, the pre-IRE PSA density was 0.12 (0.08–0.14), the median PSA nadir was 3.2 ug/L (1.4–4.2 ug/L) and the median PSA nadir level in comparison to the initial PSA was 58.00% (23.73–91.43%) 11/40 (27.5%) had significant out-of-field recurrence [8]. 

There were 12 men with high-risk PCa (six patients each for ISUP 4 and ISUP 5) who received primary IRE. The post-IRE mpMRI was low risk PI-RADS 2 in ten men and two declined follow-up mpMRI. Seven patients in this cohort proceeded to biopsy. There was no in-field recurrence (0/7, 0%) in the high-risk group, however significant out-of-field recurrence occurred in one patient and 3/7 (42.86%) had insignificant out-of-field recurrence. This of course is a very small sample size and further studies need to be performed. All 64 men treated with primary IRE remained continent, with an incontinence rate of 0%. Of the 50 men with ≥ 12 months follow-up, 28 were potent pre-operatively with 24/28 (85.71%) remaining potent after primary IRE [8]. 

Probably the largest experience with IRE come from Australia under the direction of Dr Philllip Stricker. His team recently reported a series of 229 patients with median follow up of 5 years, some patients as long as 10 years between 2013 and 2021. Follow up included PSA, MRI at 6 months and re-biopsy at 12 months [9]. Median age was 68 years old and median PSA was 5.9 ng/ml at baseline. 86% had intermediate risk and 7% had high risk. Specifically, 37 patients had USUP 3 and 14 patients had USUP 4. Overall FFS rates were 91%, 84% and 69% at 3,5 and 8 years. For USUP 3 the FFS was just over 80% and for USUP 4 was just under 80%. 24 patients had a re-do procedure of which 11 had a durable response. Metastasis free survival was 99.6%. Prostate cancer specific survival was 100% at 5 years. MRI showed successful ablation in 82% Residual cancer on repeat biopsy was found in 45 ( 24%) of patients, most of which were USUP 1. Significant cancer was detected in only 14(7.4%) in field and 31(16.3%) were out of field. Continence was close to 100% and erectile function was preserved in 87% of men. While there was a significant recurrence of repeat biopsy, the number of patients who progressed to radical whole gland therapy was low and cancer specific survival remained high. So at least at 5 years no patients were put at an increased risk of cancer death using this approach [9]. 

### Quality of Life

Focal Therapy, across various modalities, generally demonstrates better preservation of urinary and sexual function compared to radical treatments. Post-treatment continence rates generally exceed 90% and potency preservation rates above 70- 85%. Focal Therapy typically has a favorable safety profile. Reported complications are usually mild and transient, with infrequent grade 3 or higher events.

## Conclusion

It is of general consensus that patients with GG3 and higher should be considered for whole gland radical therapy including radical prostatectomy and radiation therapy. RALP, even in the best of hands, has significant quality of life issues such as ED and SUI. While most patients are able to recover adequate function with the help of medications a significant number of patients still have significantly decreased erectile function. While significant long term severe incontinence is rare, up to 15% of patients will have persistent mild (1 pad / day) SUI. Radiation therapy also has potential risk of radiation cystitis and proctitis which while rare, can be devastating when they occur. It would be desirable to be able to treat these patients with an effective, minimally invasive safe modality with decreased risks without sacrificing oncologic outcomes. When the treatment of breast cancer transitioned from radical mastectomy to lumpectomy there were similar concerns and now lumpectomy has become the mainstay of treatment for many patients with breast cancer.

### Focal Therapy Techniques

Currently the options for focal therapy ablation include HIFU, Cryotherapy, IRE, TULSA, photodynamic therapy, laser ablation and brachytherapy.

It is outside the goal of this review to advocate one modality over another. Any focal program should have multiple ablative options to tailor to patient needs. For example, patients with anterior lesions greater than 4 cm from the rectum may do better with IRE or cryotherapy since HIFU has limited ability to generate heat more than 4 cm from the probe. In addition, lesions closer to the apex may benefit from-non thermal energy sources. Patients with a J pouch would do best to avoid transrectal energy and would also do better with the trans perineal approach with IRE or cryoablation.

Numerous studies and our clinical experience is that focal therapy by almost any modality has minimal side effects with almost no SUI, minimal short lived ED of less than 20% and reduced risk of a major intra-abdominal procedure. The most common side effect would be post op urinary retention in a handful of patients. Prostate / rectal fistula is very rare. Another significant benefit is that focal therapy does not exclude patients from either repeat focal ablation or salvage therapy with either surgery or radiation in the future.

Concerns for using focal therapy for GG3 and higher grade disease is inadequate cancer control. It is well established that prostate cancer is often multifocal and bilateral. Studies looking at whole mount prostate specimens after radical prostatectomy often show bilateral disease up to 66% of men originally thought to have unilateral or focal disease [10]. 

Appropriate patient selection is the holy grail of a successful focal therapy program. If we just treat GG1 patients with low risk prostate cancer of course we will have great oncologic outcomes. You could argue that most of these patients had non lethal cancer and did not need any treatment and focal therapy would have no added benefit over active surveillance. On the other end of the spectrum when we treat patients with high risk disease our outcomes will be less favorable. The key is to identify patients with focal intermediate risk disease who have no evidence of metastatic disease or locally advanced disease such as seminal vesicle invasion or extracapsular extension.

MRI, saturation biopsy and PSMA PET scan have both greatly enhanced our ability to stage prostate cancer both for local aggressiveness as well as metastatic disease. Any patient with either would not be a good candidate for focal therapy. In addition, MRI is often able to identify an index lesion.

Clearly the best candidate for focal therapy would be a patient with a single focus of cancer in a suspicious MRI lesion on fusion biopsy without evidence of locally advanced disease. In addition, any patient with cancer confined to one side of the prostate is a candidate for a hemi-ablation. Lastly, patients with a GG2 or GG3 lesion on one side but a low volume GG1 lesion on the contralateral side are also reasonable candidate for focal ablation using the argument that the index lesion drives the prognosis and by treating the higher grade index lesion and leaving the lower grade contralateral lesion (that you would normally observe ) you can adequately treat the patient w focal ablation while maintaining quality of life. All patients who undergo focal therapy need close follow up with PSA, repeat mpMRI and biopsy.

Lastly, focal therapy does not preclude patients from either repeat focal therapy in the future or salvage whole gland therapy with either surgery or radiation therapy.

### Patient follow-up after Focal Therapy

PSA is notoriously inaccurate for following patients after focal therapy. The reason for this is that so much of the normal prostate is being untreated. Most centers include serial repeat mpMRI and bx at 6–12 months and then for cause after. It is challenging to define what is a focal therapy failure and how we should judge these treatments in terms of success. As stated above PSA response alone is often inadequate and follow up MRI and biopsy is crucial for following these patients. Is in field recurrence a failure? Is a rising PSA a failure? Or should we be looking at failure free survival (FFS) meaning should we be more concerned with the rates of needing salvage therapy and death coupled with disease specific and overall survival as a better measure of success. If a patient has a low grade recurrence in field or out of field but never progresses to needing salvage therapy or death from prostate cancer then the treatment was a success while maintaining high quality of life.

### Clinical Trials

There are multiple ongoing trials looking at the efficacy of Focal Therapy compared to whole gland therapy and active surveillance. CHRONOS is a phase 2 trial in men with newly diagnosed intermediate to high risk prostate cancer. Arm A patients are randomized to whole gland therapy (RALP, XRT, Brachytherapy) vs. Focal therapy with either HIFU or Cryotherapy. Arm B FT alone vs. FT w neoadjuvant. Finasteride or Bicalutamide.

FOCUS Trial is an ongoing multicenter registry of MRI-guided focused ultrasound focal therapy for patients with intermediate-risk prostate cancer looking at 12-Month outcomes.

## Data Availability

No datasets were generated or analysed during the current study.

## References

[1] Radtke JP, Schwab C, Wolf MB, Freitag MT, Alt CD, Kesch C, et al. Multiparametric Magnetic Resonance Imaging (MRI) and MRI-Transrectal Ultrasound Fusion Biopsy for Index Tumor detection: correlation with radical Prostatectomy Specimen. Eur Urol. 2016;70(5):846–53. 10.1016/j.eururo.2015.12.052. 26810346 10.1016/j.eururo.2015.12.052

[2] Stabile A, Orczyk C, Hosking-Jervis F, Giganti F, Arya M, Hindley RG, et al. Medium-term oncological outcomes in a large cohort of men treated with either focal or hemi-ablation using high-intensity focused ultrasonography for primary localized prostate cancer. BJU Int. 2019;124(3):431–40. 10.1111/bju.14710.** Investigates FFS and re-treatment rates in large sample of HIFU patients**. 30753756 10.1111/bju.14710

[3] Johnson DC, Reiter RE. Focal therapy should not be considered for men with Gleason Grade Group 3–5 prostate Cancer. Eur Urol Focus. 2020;6(2):203–4. 10.1016/j.euf.2019.06.005. 31202786 10.1016/j.euf.2019.06.005

[4] Guillaumier S, Peters M, Arya M, Afzal N, Charman S, Dudderidge T, et al. A Multicentre Study of 5-year outcomes following focal therapy in treating clinically significant nonmetastatic prostate Cancer. Eur Urol. 2018;74(4):422–9. 10.1016/j.eururo.2018.06.006. 29960750 10.1016/j.eururo.2018.06.006 PMC6156573

[5] Khan A, Khan AU, Siref L, Feloney M. Focal cryoablation of the prostate: primary treatment in 163 patients with localized prostate Cancer. Cureus. 2023. 10.7759/cureus.37172. 37153263 10.7759/cureus.37172 PMC10162693

[6] Shah TT, Peters M, Eldred-Evans D, Miah S, Yap T, Faure-Walker NA, et al. Early-Medium-Term Outcomes of Primary Focal Cryotherapy to Treat Nonmetastatic Clinically Significant Prostate Cancer from a Prospective Multicentre Registry. Eur Urol. 2019;76(1):98–105. 10.1016/j.eururo.2018.12.030. **Multicenter review of patients who received cryotherapy and their outcomes after several years**. 30638633 10.1016/j.eururo.2018.12.030

[7] Blazevski A, Scheltema MJ, Amin A, Thompson JE, Lawrentschuk N, Stricker PD. Irreversible electroporation (IRE): a narrative review of the development of IRE from the laboratory to a prostate cancer treatment. BJU Int. 2020;125(3):369–78. 10.1111/bju.14951. 31725935 10.1111/bju.14951

[8] Yaxley WJ, Gianduzzo T, Kua B, Oxford R, Yaxley JW. Focal therapy for prostate cancer with irreversible electroporation: oncological and functional results of a single institution study. Investig Clin Urol. 2022;63(3):285–93. 10.4111/icu.20210472. 35534217 10.4111/icu.20210472 PMC9091832

[9] Scheltema MJ, Geboers B, Blazevski A, Doan P, Katelaris A, Agrawal S, et al. Median 5-year outcomes of primary focal irreversible electroporation for localised prostate cancer. BJU Int. 2023;131(Suppl 4):6–13. 10.1111/bju.15946. **Large study providing outcomes for patients with focal prostate cancer receiving IRE** . 36495481 10.1111/bju.15946

[10] Bulbul MA, El-Hout Y, Haddad M, Tawil A, Houjaij A, Bou Diab N, Darwish O. Pathological correlation between needle biopsy and radical prostatectomy specimen in patients with localized prostate cancer. Can Urol Assoc J. 2007;1(3):264–6. 10.5489/cuaj.80. 18542801 10.5489/cuaj.80 PMC2422971

